# Risk of non-alcoholic fatty liver disease in patients with chronic plaque psoriasis: an updated systematic review and meta-analysis of observational studies

**DOI:** 10.1007/s40618-022-01755-0

**Published:** 2022-02-11

**Authors:** F. Bellinato, P. Gisondi, A. Mantovani, G. Girolomoni, G. Targher

**Affiliations:** 1 Section of Dermatology and Venereology, Department of Medicine, University of Verona, Verona, Italy; 2 Section of Endocrinology, Diabetes and Metabolism, Department of Medicine, University of Verona, Verona, Italy

**Keywords:** Psoriasis, NAFLD, Meta-analysis, MAFLD, Comorbidities, Psoriatic arthritis, Metabolic syndrome

## Abstract

**Purpose:**

Chronic plaque psoriasis is associated with the presence of non-alcoholic fatty liver disease (NAFLD), but the magnitude of this association remains currently uncertain. We aimed to investigate the magnitude of the association between psoriasis and the risk of prevalent and incident NAFLD, and to assess whether psoriasis severity and/or psoriatic arthritis are associated with a greater risk of NAFLD.

**Methods:**

A systematic review and meta-analysis of observational studies evaluating the association between psoriasis and NAFLD, as diagnosed by imaging or International Classification of Diseases codes was performed. Literature search on PubMed, Scopus and Web of Science on May 3, 2021 was undertaken. Studies using liver biopsy were not available. For the meta-analysis, the random-effects modelling was adopted.

**Results:**

We identified 15 observational (case–control and cross-sectional) studies for a total of 249,933 patients with psoriasis (49% with NAFLD) and 1,491,402 controls (36% with NAFLD). Psoriasis was associated with prevalent NAFLD (*n* = 11 studies; pooled random-effects odds ratio [OR] 1.96, 95% CI 1.70–2.26; *I*^2^ = 97%, *p* < 0.01). Psoriatic patients with NAFLD had a higher mean psoriasis area and severity index (PASI) than their counterparts without NAFLD (*n* = 8 studies, pooled weighted mean difference: 3.93, 95% CI 2.01–5.84*; I*^2^ = 88%, *p* < 0.01). The risk of NAFLD was marginally higher in patients with psoriatic arthritis than in those with psoriasis alone (*n* = 5 studies, pooled random-effects OR 1.83, 95% CI 0.98–3.43; *I*^2^ = 64%, *p* = 0.03). Sensitivity analyses did not alter these findings. Funnel plot did not show any significant publication bias. A major limitation of the study was the high degree of heterogeneity across studies.

**Conclusion:**

Psoriasis is associated with prevalent NAFLD and this risk parallels the severity of psoriasis.

**Supplementary Information:**

The online version contains supplementary material available at 10.1007/s40618-022-01755-0.

## Introduction

Chronic plaque psoriasis is a common, immune-mediated, inflammatory skin disease affecting nearly 3% of adults in the general population in Western countries [1]. In the past decade, several observational studies have documented that chronic plaque psoriasis is strongly associated with multiple cardiometabolic co-morbidities, including cardiovascular disease (CVD), obesity, type 2 diabetes (T2DM) or metabolic syndrome [1, 2].

Non-alcoholic fatty liver disease (NAFLD) is considered a purely metabolic liver disease, which includes a spectrum of progressive pathologic liver conditions, ranging from simple steatosis to steatohepatitis (NASH) and cirrhosis [2]. To date, NAFLD has reached epidemic proportions and is the most common cause of chronic liver disease in Western countries (affecting up to 30% of adults in the general population and up to ~ 70% of people with T2DM) [3]. In the last decade, it has become increasingly clear that NAFLD is a ‘*multisystem’* disease [4], which is not only associated with important liver-related complications (such as liver failure or hepatocellular carcinoma [HCC]), but also with extra-hepatic diseases, such as CVD [5], chronic kidney disease (CKD) [6] and some types of extra-hepatic cancers [7].

Recently, some observational studies, although not all, have reported that individuals with chronic plaque psoriasis have an increased risk of having NAFLD compared with those without psoriasis [8]. Presently, however, the magnitude of this risk and whether the risk changes with the underlying severity of psoriasis remains uncertain. To our knowledge, there are only two small meta-analyses (published in 2015 and 2019, respectively) that have investigated the association between psoriasis and risk of prevalent NAFLD [9, 10]. These two small meta-analyses reported that psoriasis is associated with an increased risk of prevalent NAFLD, but the available data on the association of NAFLD with the severity of psoriasis or psoriatic arthritis remain inconclusive. Notably, as discussed in detail below, in the last 2 years, new observational studies examining the association between psoriasis (and its severity) and the risk of NAFLD have been published.

We undertook an updated systematic review and meta-analysis of observational studies to quantify the magnitude of the association between psoriasis and risk of prevalent and incident NAFLD, as well as to examine whether the severity of chronic plaque psoriasis or presence of psoriatic arthritis were associated with a higher risk of prevalent NAFLD.

## Methods

### Data sources and searches

We performed the systematic review according to the Preferred Reporting Items for Systematic Reviews and Meta-Analyses (PRISMA) statement [11]. Since the included studies were observational in design, we followed the reporting items proposed by Meta-analysis Of Observational Studies in Epidemiology (MOOSE) for the meta-analysis of these studies [12]. We conducted a systematic literature search from the inception date to May 3, 2021 through PubMed, Scopus and Web of Science to identify observational studies examining the risk of prevalent NAFLD amongst individuals with and without chronic plaque psoriasis. Search text terms were as follows: ((“Arthritis, Psoriatic” [Mesh]) OR (“Psoriasis” [Mesh])) AND (“Non-alcoholic Fatty Liver Disease” OR “NAFLD” [Mesh]). Searches were restricted to human studies. There were no restrictions in terms of sex, race, language, or geographic area. Additionally, we reviewed references from relevant original papers and review articles to identify further eligible studies not covered by the original database searches.

### Study selection

Studies were included in the meta-analysis if they meet the following criteria: (1) observational studies examining the association between chronic plaque psoriasis and risk of NAFLD; (2) studies reporting odds ratios (ORs) with 95% confidence intervals (95% CIs) values for the outcome of interest; (3) studies in which the diagnosis of both psoriasis and psoriatic arthritis were based on clinical examinations and specific international criteria, namely CASPAR criteria [13]; (4) studies in which the psoriasis severity was estimated using the psoriasis area and severity index (PASI); and (5) studies in which the diagnosis of NAFLD was based on liver biopsy, imaging techniques or International Classification of Diseases, 9th Revision (ICD-9) or ICD-10 codes, in the absence of significant alcohol consumption or other competing causes for hepatic steatosis [14].

Criteria for exclusion of the studies from the meta-analysis were as follows: (1) congress abstracts, case reports, theses, reviews, commentaries, editorials, or practice guidelines; (2) studies not reporting ORs and 95% CIs for the outcome of interest; and (3) studies conducted in paediatric population (< 18 years).

### Data extraction and quality assessment

Data from studies eligible for the aggregate data meta-analysis were extracted by two authors independently (FB and PG). Any disagreements were resolved by consensus and a third author if needed (GG).

For all eligible studies, we extracted information on publication year, study design, study country, sample size, population characteristics, methods used for the diagnosis of psoriasis and NAFLD, psoriasis severity assessed by PASI score, psoriatic arthritis (PsA), matching and confounding factors included in multivariable regression analyses. In case of multiple publications, we included the most up-to-date or comprehensive information. We did not contact any corresponding author of the eligible studies in order to obtain additional information for the meta-analysis.

Two authors (FB and PG) independently assessed the risk of bias. Any discrepancies were addressed by a re-evaluation of the original article by a third author (GG). Since the eligible studies were non-randomized, the Newcastle–Ottawa Scale (NOS) was used to judge the quality of the studies included in the meta-analysis, as recommended by the Cochrane Collaboration. Briefly, the NOS uses a star system to evaluate a study in three domains: selection of participants, comparability of study groups, and ascertainment of outcomes of interest. We used an adapted version of the NOS for cross-sectional studies. We judged studies that received a NOS score of at least 8 stars to be at low risk of bias, thereby reflecting the highest quality [15].

### Data synthesis and analysis

The primary outcome measure of the meta-analysis was the risk of having NAFLD in patients with chronic plaque psoriasis and non-psoriatic control subjects. The ORs with 95% CIs were considered as the effect size for all eligible studies. When studies had several adjustment models, we extracted those that reflected the maximum extent of adjustment for potentially confounding risk factors. The adjusted ORs of all eligible studies were then pooled, and an overall estimate of effect size was calculated using the DerSimonian–Laird random-effects model. The psoriasis severity, as assessed by PASI score, in patients with and without NAFLD was displayed as weighted mean difference (WMD) and 95% CIs for the changes of mean PASI score between psoriatic patients with and without coexisting NAFLD. Visual inspection of the forest plot was used to investigate the possibility of statistical heterogeneity. Statistical heterogeneity was also assessed by the *I*^2^-statistics, which provides an estimate of the percentage of variability across studies that is due to heterogeneity rather than chance alone. According to Higgins and Thompson [16], *I*^2^-values of approximately 25% represent low heterogeneity; approximately 50% represent medium heterogeneity; and approximately 75% represent high heterogeneity. Publication bias was evaluated using the funnel plot, the Begg’s rank correlation test [17] and the Egger’s regression test [18].

To explore the possible sources of heterogeneity among the eligible studies and to test the robustness of the observed associations, we performed subgroup analyses stratifying the eligible studies by study country, methodology used for the diagnosis of NAFLD, NOS scale (i.e. the ‘high-quality’ studies), or whether they had adjustment at least for age, sex and body mass index (BMI]. Additionally, we performed univariable meta-regression analyses to evaluate the impact of specific moderators (i.e., age, sex and BMI) on the effect size of the risk of having psoriasis-related NAFLD across the eligible studies. We also tested for possible excessive influence of individual studies using a meta-analysis influence test that eliminated each of the included studies one at a time. All statistical tests were two sided and used a significance level of *p* < 0.05. We used Review Manager version 5.3 (Copenhagen: The Nordic Cochrane Centre, The Cochrane Collaboration, 2014) and STATA® software v16.1 (StataCorp, College Station, Texas, USA) for all statistical analyses. This systematic review and meta-analysis is registered in PROSPERO, number: CRD42021247549.

## Results

### Characteristics of the included articles

**Fig. 1:**
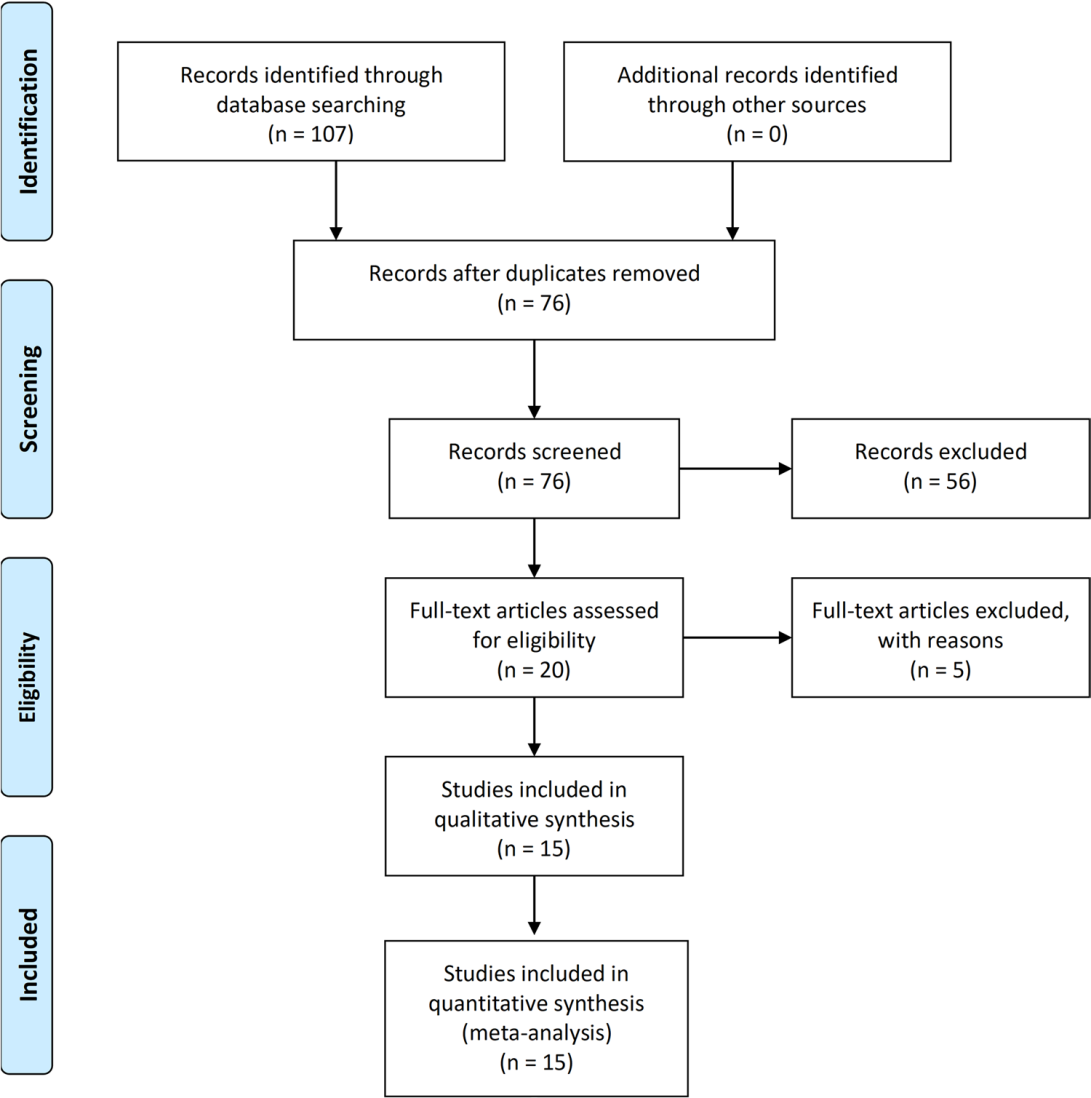
PRISMA flow diagram describing the results of the literature research and study selection

From a total of 76 retrieved articles (after excluding duplicates), a total of 20 eligible studies from PubMed, Scopus or Web of Science databases were identified based on the titles and abstracts, (kappa agreement = 90%) (Fig. 1). After full text examination of these 20 potentially eligible studies, we excluded five studies because unsatisfactory inclusion criteria or unacceptable outcome measures, (kappa agreement = 90%) (Supplementary Table 1). After of this exclusion, a total of 15 observational studies were analysed in the meta-analysis. Of these 15 eligible studies, 11 studies were included in the pooled primary analysis that compared the risk of prevalent NAFLD between psoriatic patients and non-psoriatic controls; 8 studies were included in the analysis examining the association between NAFLD and psoriasis severity, as assessed by PASI score; 5 studies were included in the secondary analysis assessing the risk of prevalent NAFLD among psoriatic patients with and without PsA [19–33]. Of note, the study by Ogdie et al. included both a cross-sectional and a cohort design [24].

In particular, the main characteristics of the 11 eligible studies assessing the risk of prevalent NAFLD are reported in Table 1. Overall, these cross-sectional or case–control studies had aggregate data on 249,933 psoriatic patients (mean age 55 years, mean BMI 27.7 kg/m^2^, 54% were men, 49% had NAFLD) and 1,491,402 non-psoriatic healthy controls (mean age 53 years, mean BMI 26.7 kg/m^2^, 49% were men, 36% had NAFLD). Four studies were carried out in the Europe (Italy and the Netherlands), 3 studies were carried out in Asia (Iran and Taiwan) and 4 studies were carried out in the United States. NAFLD was diagnosed by ultrasonography in 7 studies and by ICD-9/10 codes in 4 studies. No studies with liver biopsy data were available for the meta-analysis.

**Table 1:** Characteristics of the eligible observational studies assessing the risk of prevalent and incident NAFLD in patients with and without psoriasis

References (PMID)	Country	Psoriasis patients, *n*	Healthy controls, *n*	Age, years^a^	PASI^a^	Diagnosis of NAFLD	Crude risk of NAFLD (OR 95% CI)	Covariate adjustment	Adjusted risk of NAFLD (OR 95% CI)	Risk of NAFLD in PsA vs. PsO (OR 95% CI)
Gisondi et al. [21] (19560226)	Italy	130	260	51.2 ± 13.4	NR	US	–	Age, sex, BMI (matched)	2.26 (1.46–3.51)	1.02 (0.51–2.03)
Tsai et al. [25] (21543188)	Taiwan	51,800	207,200	47.5 ± 16.4	NR	ICD	NR	Age, sex, urbanization level	2.27 (1.90–2.71)	NR
Madanagobalane and Anandan [23] (22672067)	India	333	330	46.26 ± 11.5	5.16 ± 6.06	US and TE	NR	Age, sex, BMI (matched)	2.22 (1.36–3.63)	3.71 (1.89–7.30)
van der Voort et al. [26] (24373781)	Netherlands	118	2174	76.2 ± 6.0	2.9 ± 2.8	US	1.70 (1.17–2.46)	^c^	1.70 (1.13–2.58)	NR
Abedini et al. [19] (25958919)	Iran	123	123	43 (30–56)^b^	9 (4–16)^b^	US	–	Age, sex, BMI	3.59 (2.12–6.07)	NR
Gisondi et al. [22] (26537011)	Italy	124	79	55 ± 12	13 ± 10	US	2.20 (1.19–4.06)	NA	NR	1.00 (0.52–1.95)
van der Voort et al. [27] (26062958)	Netherlands	74	1535	71.2 ± 6.5	2.0 (3.2)^b^	US and TE	1.07 (0.63–1.72)	NA	NR	NR
Awosika et al. [20] (29942422)	United States	101	51	44.2 ± 13.6	NR	US	3.08 (1.00–9.53)	Age, sex, BMI	2.63 (0.51–13.6)	NR
Ogdie et al. [24] (29104161)	United States	*Cross-sectional study* 4539 (mild PsO)	87,596	45.67 ± 11	NR	ICD	NR	Age, sex	1.29 (0.92–1.81)	NR
		3133 (moderate PsO)		45.27 ± 11					1.32 (0.89–1.97)	
		*Longitudinal study* 1088 (severe PsO)		44.49 ± 11					1.28 (0.66–2.48)	
		592 (mild PsO)	3,654	NR			1.18 (1.08–1.28)^g^	^d^	1.18 (1.07–1.30)^g^	
		75 (moderate to severe PsO)		NR			3.09 (2.46–3.88)^g^		2.23 (1.73–2.87)^g^	
Yousaf et al. [29] (32965553)	United States	NR	NR	NR	NR	ICD	NR	^e^	2.22 (2.21–2.23)	NR
Yang et al. [28] (33722548)	United States	14,593	1,935,906	NR	NR	ICD	NR	^f^	1.92 (1.83–2.01)	NR

*PMID* PubMed Identifier, *US* ultrasonography, *TE* transitional elastography, *ICD* International Classification of Diseases codes, *NA* not applicable, *NR* not reported, *OR* odds ratio, *PASI* Psoriasis Area Severity Index, *PsA* psoriatic arthritis, *PsO* psoriasis

^a^Mean ± standard deviation

^b^Median (IQR) interquartile range

^c^Age, sex, alcohol consumption, pack-years and smoking status, metabolic syndrome, alanine aminotransferase

^d^Age at start date, sex, smoking, alcohol intake, body mass index category, and use of oral glucocorticoids and NSAID in the baseline period

^e^Age, sex, race, income, insurance status, obesity, smoking, and alcohol consumption

^f^Age, sex, income quartile, race, smoking, alcoholism, metabolic syndrome, HIV/AIDS, and type 2 diabetes

^g^Hazard ratio

The main characteristics of the 8 eligible studies examining the association between NAFLD and psoriasis severity, as assessed by PASI score, are reported in Table 2. Of these 8 studies, 4 recruited European individuals, 2 studies involved Asian subjects and 2 studies involved United States individuals. NAFLD was diagnosed by imaging techniques (mostly ultrasonography) in all eligible studies, although 2 ones also used biopsy in a small subset of psoriatic patients (for a total of 57 cases).

**Table 2:** Characteristics of the eligible studies evaluating the psoriasis severity (assessed by PASI score) in psoriatic patients with and without NAFLD

References (PMID)	Country	Patients, *n*	Age, years^b^	PASI^b^	Diagnosis of NAFLD	Prevalence of NAFLD, *n* (%)	Risk of NAFLD in PsA vs. PsO (OR 95% CI)	Mean PASI difference in patients with NAFLD (IV, 95% CI)^a^
Gisondi et al. [21] (19560226)	Italy	130	51.2 ± 13.4	13.1 ± 11.0	US	61 (47)	1.02 (0.51–2.03)	7.90 (4.67–11.13)
Miele et al. [31] (19664838)	Italy	84	50.1 ± 15	16.4 ± 10.0	US, biopsy (5 cases)	84 (59.2)	3.53 (0.98–12.77)	2.00 (− 1.96 to 5.96)
Madanagobalane and Anandan [23] (22672067)	India	333	46.3 ± 11.5	6.5 ± 10.8	US and TE	58 (17.4)	3.71 (1.89–7.30)	2.20 (− 0.62 to 5.02)
Roberts et al. [33] (25521607)	United States	103	52.7 ± 12	5.2 ± 4.9	TE, biopsy (52 cases)	48 (46.6)	NR	2.21 (0.64–3.79)
Gisondi et al. [22] (26537011)	Italy	124	55.0 ± 12.0	13.0 ± 10.0	US	55 (44)	1.00 (0.52–1.95)	8.40 (5.14–11.66)
Narayanasamy et al. [32] (28053681)	India	250	44.7 ± 12.0	27.8 ± 13.5	US and TE	113 (45.2)	NR	9.69 (6.29–13.09)
Awosika et al. [20] (29942422)	United States	101	44.2 ± 13.6	4.8 ± 2.1	US	21 (21.2)	NR	0.72 (−0.20 to 1.64)
Magdaleno Tapial et al. [30] (31731325)	Spain	71	46.7 ± 14	14.4 ± 6.5	US and TE	37 (52.1)	2.15 (0.64–7.21)	1.30 (0.30–2.30)

*NR* not reported, *OR* odds ratio, *US* ultrasonography, *IV* interval variable, *TE* transitional elastography, *PASI* Psoriasis Area Severity Index, *PsA* psoriatic arthritis

^a^Median (IQR)

^b^Mean ± standard deviation

### Risk of prevalent NAFLD in patients with and without chronic plaque psoriasis

**Fig. 2:**
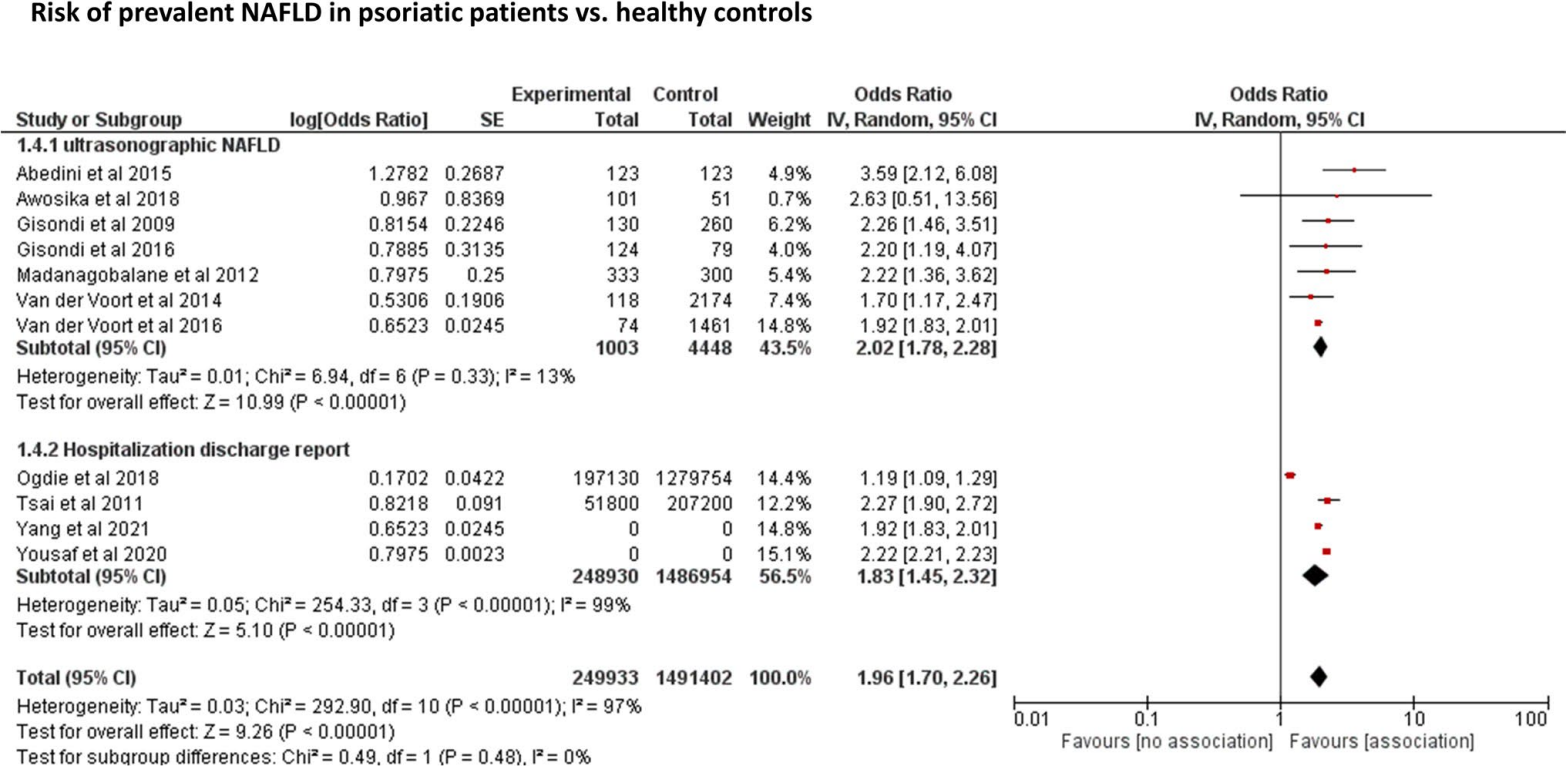
Forest plot and pooled estimates of the effect of chronic plaque psoriasis on the risk of prevalent NAFLD in 11 eligible studies, stratified by NAFLD diagnosis (ultrasonography vs. International Classification of Diseases codes)

The distribution of the 11 eligible studies by estimate of the association between presence of psoriasis and risk of NAFLD, stratified by methods used for NAFLD diagnosis (ultrasonography *vs*. ICD codes), is plotted in Fig. 2. Overall, patients with chronic plaque psoriasis had an approximately doubled odds ratio for NAFLD compared to non-psoriatic control subjects (pooled random-effects OR 1.96, 95% CI 1.70–2.26, *I*^2^ = 97%, *p* < 0.001). Similar results were observed when the studies were stratified by the methods of NAFLD diagnosis, i.e., either by ultrasonography (*n* = 7; pooled random-effects OR 2.02, 95% CI 1.78–2.28, *I*^2^ = 13%, *p* = 0.33) or by ICD codes (*n* = 4; pooled random-effects OR 1.83, 95% CI 1.45–2.32, *I*^2^ = 97%, *p* < 0.001). Notably, as shown in the figure, there was no significant heterogeneity across the eligible studies that used ultrasonography to diagnose NAFLD (*I*^2^ = 13%).

### Psoriasis severity in patients with and without NAFLD

**Fig. 3:**
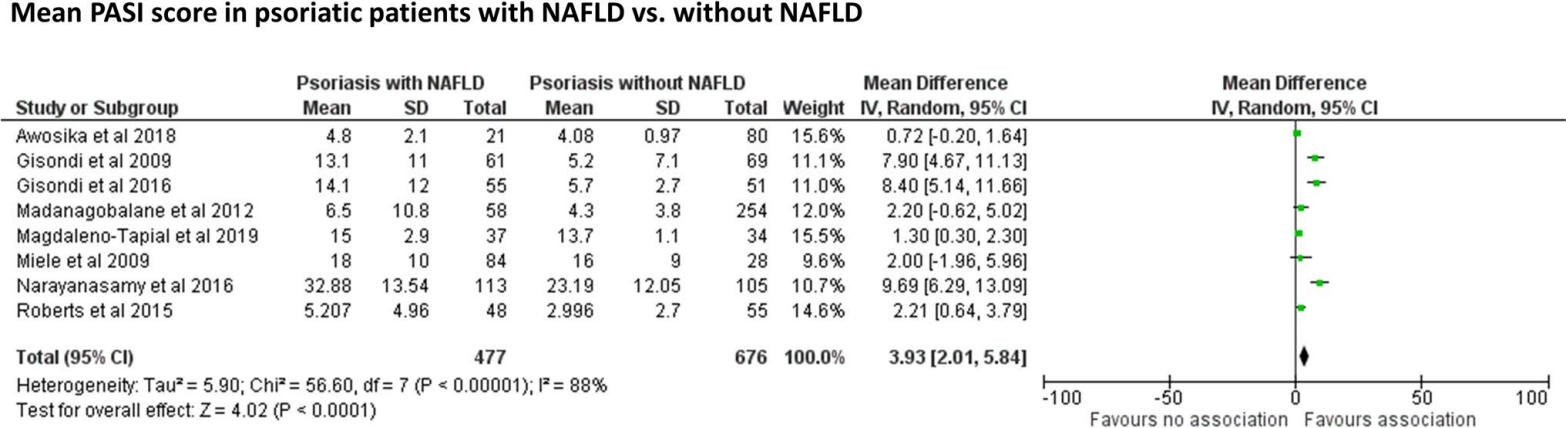
Forest plot and pooled estimates of PASI score in psoriatic patients with and without NAFLD (*n* = 8 studies included). Data are expressed as weighted mean difference (WMD) and 95% confidence intervals

Figure 3 shows the pooled estimates of mean PASI score among psoriatic patients with and without coexisting NAFLD across 8 eligible studies. Psoriatic patients with NAFLD had significantly greater mean PASI score than their counterparts without NAFLD (pooled WMD: 3.93, 95% CI 2.01–5.84; *I*^2^ = 88, *p* < 0.0001).

### Risk of incident NAFLD in patients with psoriasis

In a subgroup of patients, the study conducted by Ogdie et al. also assessed the risk of incident NAFLD in patients with psoriasis or in those with other autoimmune diseases. In particular, in this study that involved 197,130 patients with psoriasis, 12,308 patients with PsA, 54,251 patients with rheumatoid arthritis and 1,279,754 healthy controls, Ogdie et al*.* showed that compared to healthy controls, patients with psoriasis had a higher risk of developing NAFLD (as detected by ultrasonography) over a mean follow-up period of nearly 10 years, even after adjustment for age, sex, BMI and other metabolic risk factors. Notably, this risk increased across severity of psoriasis with an adjusted-hazard ratio of 1.18 [95%CI 1.07–1.30] for patients with mild psoriasis and adjusted-hazard ratio of 2.23 [95%CI 1.73–2.87] for patients with moderate to severe psoriasis, respectively [24].

### Risk of prevalent NAFLD in patients with and without psoriatic arthritis

**Fig. 4:**
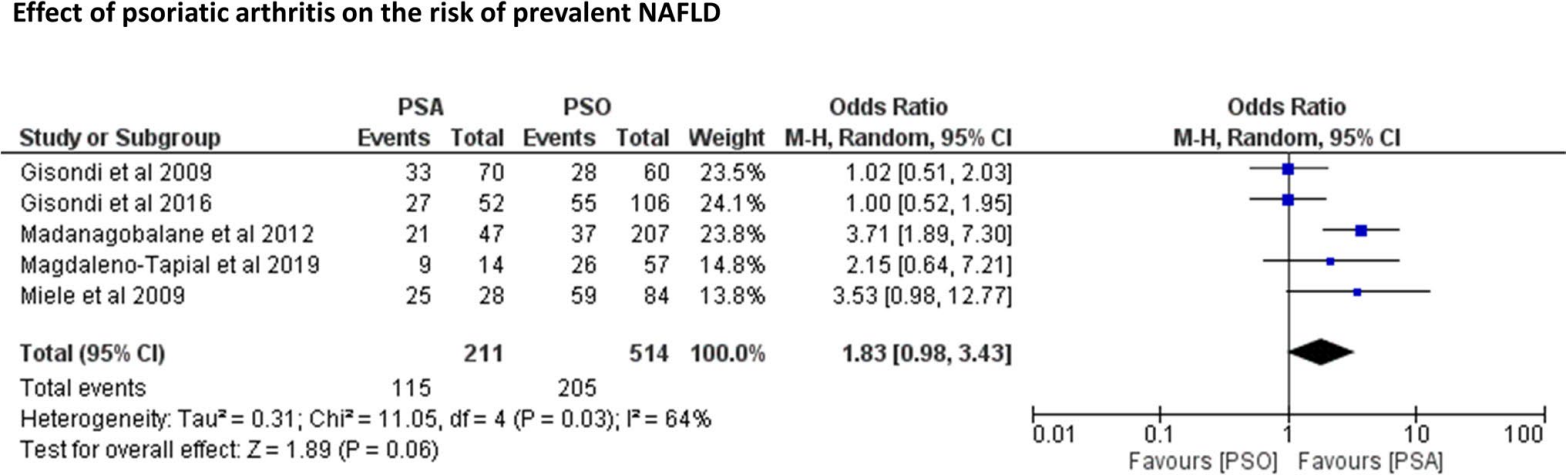
Forest plot and pooled estimates of the effect of psoriatic arthritis (PsA) on the risk of prevalent NAFLD (*n* = 6 studies included)

Figure 4 shows the risk of prevalent NAFLD in psoriatic patients with and without coexisting PsA across 5 eligible studies (including a total of 725 psoriatic patients, 514 of whom had PsA). Patients with PsA tended to have a higher risk of NAFLD compared to those without PsA (pooled random-effects OR 1.83, 95% CI 0.98–3.43; *I*^2^ = 64%, *p* = 0.061), but this difference did not reach statistical significance.

### Subgroup analyses and meta-regressions

Subgroup analyses were performed to investigate potential causes of heterogeneity across the 11 eligible articles. The stratification of the articles by study country (Supplementary Fig. 1), NOS quality scale (Supplementary Fig. 2), or degree of covariate adjustment (Supplementary Fig. 3) did not influence the association between psoriasis and risk of NAFLD.

We also examined the potential excessive impact of individual studies through an analysis that excluded each of the included studies one at a time (Supplementary Fig. 4). The exclusion of each of the studies from the pooled primary analysis did not affect the overall risk of NAFLD.

Finally, Supplementary Fig. 5 shows the results of univariable meta-regressions. These analyses did not show any significant effects of age (*β* = − 0.01, 95% CI − 0.03 to 0.1, *R*^2^ = 33.65%), male sex (*β* = 0.01, 95% CI − 0.02 to 0.04, *R*^2^ = − 41.25%), or body mass index (*β* = 0.12, 95% CI − 0.18 to 0.42, *R*^2^ = 1.36%) (panels A–C) on the association between chronic plaque psoriasis and risk of having NAFLD.

### Risk of bias

As shown in Supplementary Fig. 6, the ranking on the NOS was higher than eight points (i.e., low risk of bias) in four studies, equal to seven points (i.e. medium risk of bias) in 2 studies, and six or fewer points in the remaining 5 studies (i.e. high risk of bias). Publication bias was estimated unlikely because no asymmetry of the funnel plots was found, as formally tested by the Begg’s rank correlation test (*p* = 0.436), or the Egger’s regression test (*p* = 0.183) (Supplementary Fig. 7).

## Discussion

Our updated meta-analysis provides evidence for a significant association between chronic plaque psoriasis and risk of prevalent NAFLD, as detected by ultrasonography or ICD-9/10 codes. Our meta-analysis involves a total of 15 observational cross-sectional studies with aggregate data on more than 1.7 million individuals, i.e., 249,933 psoriatic patients (49% with NAFLD) and 1,491,402 non-psoriatic healthy controls (36% with NAFLD). Specifically, we found that patients with chronic plaque psoriasis had a nearly twofold higher odds of prevalent NAFLD compared to non-psoriatic healthy controls. The magnitude of this risk remained essentially unchanged even when the comparison was stratified by study country, modality of NAFLD diagnosis, NOS quality scale or degree of covariate adjustment. Notably, the risk of NAFLD appeared to increase further with the severity of psoriasis, given that psoriatic patients with NAFLD had significantly higher mean PASI score than their counterparts without NAFLD. Furthermore, the risk of NAFLD also tended to be higher among patients with PsA than among those with skin psoriasis alone.

Our findings corroborate and extend the results of two previous smaller meta-analyses (published in 2015 and 2019) [9, 10]. These two meta-analyses included a lower number of observational studies (from 6 to 9 cross-sectional or case–control studies) with a smaller overall sample size. In the first meta-analysis including 6 case–control studies, Candia et al*.* reported that psoriatic patients had 2.1-fold increased odds of prevalent NAFLD (as detected by ultrasonography or ICD-9/10 codes) compared to non-psoriatic individuals (*n* = 6 studies included; pooled random-effects OR 2.15, 95% CI 1.57–2.94; *I*^2^ = 74%) [9]. This risk appeared to be comparable between patients with mild psoriasis and those with moderate to severe psoriasis. In second meta-analysis that incorporated 9 cross-sectional or case–control studies, Phan et al. also showed an almost doubled risk of having NAFLD (as detected by ultrasonography or ICD-9/10 codes) in patients with psoriasis compared to non-psoriatic controls (*n* = 6 studies included; pooled random-effects OR 1.95, 95% CI 1.35–2.83; *I*^2^ = 91%) [10]. In this meta-analysis, the authors failed to show any significant association between the severity of psoriasis (assessed by PASI score) and NAFLD, although the odds of NAFLD was marginally (but not significantly) higher in patients with PsA than in those with skin psoriasis alone (*n* = 3 studies; pooled random-effects OR 3.74, 95% CI 0.85–16.3; *I*^2^ = 54%).

Collectively, therefore, compared to the aforementioned two smaller meta-analyses, we have almost doubled the number of eligible studies, increased the overall sample size, and performed subgroup analyses and meta-regressions to test the robustness of the observed associations. That said, the evidence from this and the two previously published meta-analyses supports the existence of a significant association between the presence and severity of psoriasis and the risk of prevalent NAFLD.

To date, the precise underlying mechanisms linking chronic plaque psoriasis and NAFLD are complex and poorly understood. Psoriasis and NAFLD may share complex, multi-factorial and overlapping metabolic abnormalities. A cluster of interconnected features defining the metabolic syndrome are known to be closely associated with chronic plaque psoriasis, including abdominal obesity, atherogenic dyslipidaemia, hypertension, insulin resistance and dysglycaemia [34]. In particular, insulin resistance plays a pathogenic role in the development of NAFLD, as it promotes an increased free fatty acid flux from visceral adipose tissue into the liver, thereby increasing hepatic de novo lipogenesis [34]. On the other hand, hepatic fat accumulation may further aggravate systemic and hepatic insulin resistance and promote increased hepatic glucose production [35]. Additionally, overlapping mechanisms of the so-called “metaflammation” may also contribute to the complex link between psoriasis and NAFLD. Indeed, we found that both psoriasis severity and PsA correlated with an increased risk of prevalent NAFLD. The secretion of pro-inflammatory, pro-thrombotic and oxidative stress mediators in both psoriatic skin and adipose tissue might act systemically and promote insulin resistance and other metabolic derangements that promote the development and progression of NAFLD. For example, tumour necrosis factor-alfa, as well as interleukins (IL)-1, IL-2, IL-6 and IL-17 may contribute to psoriatic plaque development, as well as impaired glucose metabolism and insulin resistance in both hepatocytes and adipocytes [36]. However, the link between NAFLD and psoriasis is more complex than previously believed. Indeed, it is also possible to hypothesize that NAFLD (especially in its more advanced forms) may release multiple pro-inflammatory and pro-oxidant mediators (which is also one of the most important mechanisms by which NAFLD may contribute to the development of CVD and other extra-hepatic complications) [4–7] that may adversely influence the severity of psoriasis by increased keratinocyte proliferation, increased inflammation, and up-regulation of various vascular adhesion molecules [8].

We believe that the observed association between psoriasis and NAFLD is clinically relevant and may have practical implications. Whilst phototherapy or topical treatments are not usually associated with significant abnormalities in metabolic parameters and serum liver enzymes, specific pharmacological treatments may, instead, adversely affect metabolic comorbidities, including NAFLD. In this regard, it is also important to highlight that patients with moderate-to-severe psoriasis are often treated with potentially hepatotoxic drugs, such as methotrexate or cyclosporine. Methotrexate may induce histological changes in the liver that are comparable to those observed in NAFLD [37, 38]. In addition, psoriatic patients with type 2 diabetes or obesity are at higher risk of developing hepatic fibrosis during methotrexate treatment compared with their counterparts without coexisting metabolic comorbidities [8]. Cyclosporine is metabolized by the hepatic cytochrome P450 system and may worsen glycaemic control in patients with type 2 diabetes, exacerbate arterial hypertension and predispose to atherogenic dyslipidemia [39]. For this reason, cyclosporine should be used cautiously in psoriatic patients with NAFLD or other metabolic comorbidities. Conversely, the newer biologic drugs do not seem to affect negatively liver steatosis and metabolic parameters, although a weight gain has been reported with the long-term use of some TNF-α antagonists [40]. IL-17 inhibitors are being investigated as a potential therapeutic targeting therapy for patients with NAFLD and psoriasis [41]. Consequently, we believe that the presence of NAFLD in psoriatic patients should be always considered when choosing pharmacological treatment, as some drugs for psoriasis may be potentially hepatotoxic.

Our findings also imply that psoriatic patients might be screened with liver ultrasonography especially when there are also coexisting metabolic features associated with NAFLD (e.g., obesity, atherogenic dyslipidemia or dysglycemia). However, the optimal method of screening in these patients is currently unknown. In view of the intrinsic limitations of measurement of serum liver enzymes alone as initial screening test for NAFLD, we believe that liver ultrasonography combined with the use of non-invasive biomarkers of advanced fibrosis (such as NAFLD fibrosis score [NFS] or Fibrosis-4 score [FIB-4]) or, alternatively, vibration-controlled transient elastography (Fibroscan) might be fruitful as first-line choice in identifying psoriatic patients with NAFLD and advanced fibrosis, in order to refer to a hepatologist [8]. In addition, we also believe that all psoriatic patients with NAFLD should be also followed routinely to assess the development of liver-related, metabolic and CVD complications [8].

This meta-analysis has some important limitations that should be mentioned. First, the cross‐sectional design of the studies precludes us from establishing causality and temporality for the observed associations. Second, the overall quality of the included studies is not consistently high and some of the eligible studies reported incomplete adjustments for potential confounding factors, thus the possibility of residual confounding by unmeasured factors cannot be ruled out. The degree of adjustment performed in most of the eligible studies is limited, as some studies did not adjust for BMI and, most importantly, no studies adjusted also for insulin resistance or plasma inflammatory biomarkers. Thirdly, even though we used the DerSimonian–Laird random-effects model, the interpretation of some results of this meta‐analysis should require some caution in view of the high heterogeneity observed. However, it is important to underline that we found a very low heterogeneity (*I*^2^ = 13%) when we pooled the studies examining the association between psoriasis and risk of NAFLD, where the diagnosis of NAFLD was made with ultrasonography. Another potential limitation of our meta‐analysis is that the majority of the eligible studies used imaging techniques (mostly ultrasonography) or ICD-9/10 codes that are not always reported by physicians during their daily clinical practice. Indeed, only two studies used liver biopsy, which is the reference method for diagnosing and staging NAFLD, but exclusively in a number of patients that was too small for making any statistical analysis. Thus, it is currently uncertain if psoriatic patients are also more likely to have more advanced forms of NAFLD (i.e., NASH with varying levels of fibrosis) compared with non-psoriatic controls.

Although these limitations, our meta-analysis has some important strengths. This review summarizes data from large studies that are likely to reflect people with chronic plaque psoriasis usually seen in clinical practice. We could not definitely exclude a selective reporting bias, but we believe that our search has made such bias unlikely. Finally, visual inspection of the funnel plot did not show any publication bias.

In conclusion, this updated meta-analysis of more than 1.7 million individuals of different countries shows that psoriasis is significantly associated with a nearly twofold increased odds of prevalent NAFLD. This risk parallels the underlying severity of chronic plaque psoriasis. Given the observational design of the eligible studies and the fact that most eligible studies used ultrasonography for detecting NAFLD, the findings of this meta-analysis pave the way for novel large, prospective, histologically based studies. Mechanistic studies are also needed to better elucidate the existing but complex link between psoriasis and NAFLD.

## Supplementary Information

Below is the link to the electronic supplementary material.

Supplementary file1 (DOCX 13 kb)

Supplementary figure 1. Subgroup analysis. Forest plot and pooled estimates of the effect of psoriasis on the risk of NAFLD in 11 eligible studies, stratified by study country. (TIF 4129 kb)

Supplementary figure 2. Subgroup analysis. Forest plot and pooled estimates of the effect of psoriasis on the risk of NAFLD in 11 eligible studies, stratified by NOS quality scale. (TIF 3928 kb)

Supplementary figure 3. Subgroup analysis. Forest plot and pooled estimates of the effect of psoriasis on the risk of NAFLD in 11 eligible studies, stratified by degree of covariate adjustment. (TIF 4473 kb)

Supplementary figure 4. Meta-analysis estimates, given named study is omitted (for the studies included in Figure 2). (TIF 1170 kb)

Supplementary figure 5. Univariable meta-regression analyses to examine the impact of age (panel A), male sex (panel B), or body mass index (panel C) on the effect size of the risk of psoriasis-related NAFLD. (TIF 2172 kb)

Supplementary figure 6. Risk of bias summary for each eligible study assessed by the Cochrane Collaboration’s tool. (TIF 3680 kb)

Supplementary figure 7. Funnel plot of standard error by log-odds ratio for the risk of NAFLD (for the 11 eligible studies included in Figure 2). P-values by the rank correlation Begg’s test. (TIF 1848 kb)

## Data Availability

Not applicable.
