# Supplementary material for: Risk of non-alcoholic fatty liver disease in patients with chronic plaque psoriasis: an updated systematic review and meta-analysis of observational studies
Source: J Endocrinol Invest. 2022 Feb 11;45(7):1277–88. doi: 10.1007/s40618-022-01755-0 (PMC9184411; doi:10.1007/s40618-022-01755-0)
Supplement: Supplementary file 1 — Supplementary file1 (DOCX 13 kb) [file 40618_2022_1755_MOESM1_ESM.docx]

**Supplementary Table 1. Excluded studies at the eligibility step as reported in the PRISMA flow diagram (n=5 studies).**

| **Author, year (PMID)** | **Main Reason** |
| --- | --- |
| Campanati A et al. 2013 (23065020) | Unsatisfactory outcome measures |
| Pongpit J et al. 2016 (27006950) | Unsatisfactory outcome measures |
| Xu X et al. 2017 (28099469) | Unsatisfactory outcome measures |
| Cheng HS et al. 2018 (29785393) | Unsatisfactory inclusion criteria |
| Gadha N et al. 2020 (33117662) | Unsatisfactory outcome measures |
