# Supplementary figures and images for: Risk of non-alcoholic fatty liver disease in patients with chronic plaque psoriasis: an updated systematic review and meta-analysis of observational studies
Source: J Endocrinol Invest. 2022 Feb 11;45(7):1277–88. doi: 10.1007/s40618-022-01755-0 (PMC9184411; doi:10.1007/s40618-022-01755-0)

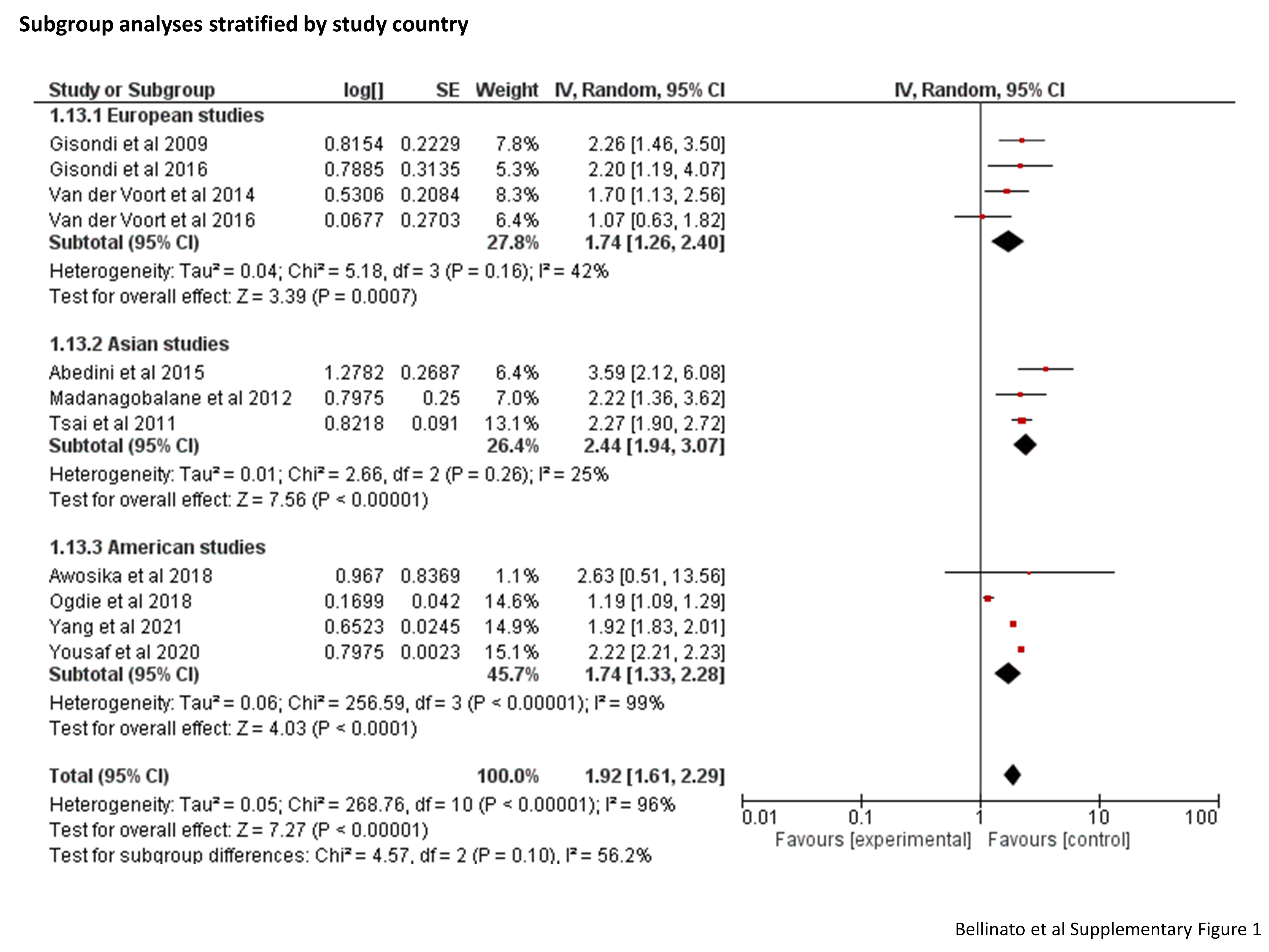

Supplement: Supplementary file 2 — Supplementary figure 1. Subgroup analysis. Forest plot and pooled estimates of the effect of psoriasis on the risk of NAFLD in 11 eligible studies, stratified by study country. (TIF 4129 kb) [file 40618_2022_1755_MOESM2_ESM.tif]

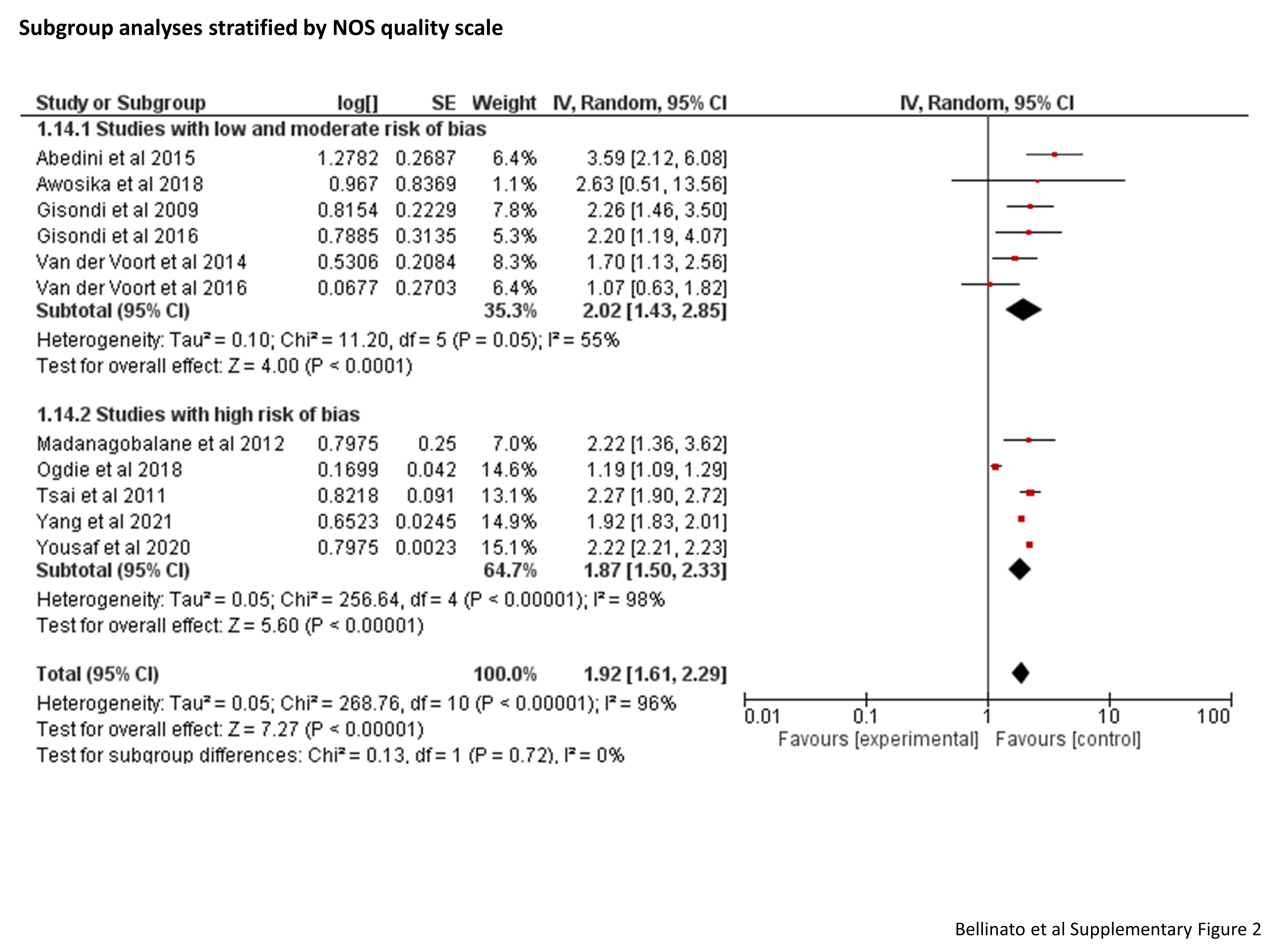

Supplement: Supplementary file 3 — Supplementary figure 2. Subgroup analysis. Forest plot and pooled estimates of the effect of psoriasis on the risk of NAFLD in 11 eligible studies, stratified by NOS quality scale. (TIF 3928 kb) [file 40618_2022_1755_MOESM3_ESM.tif]

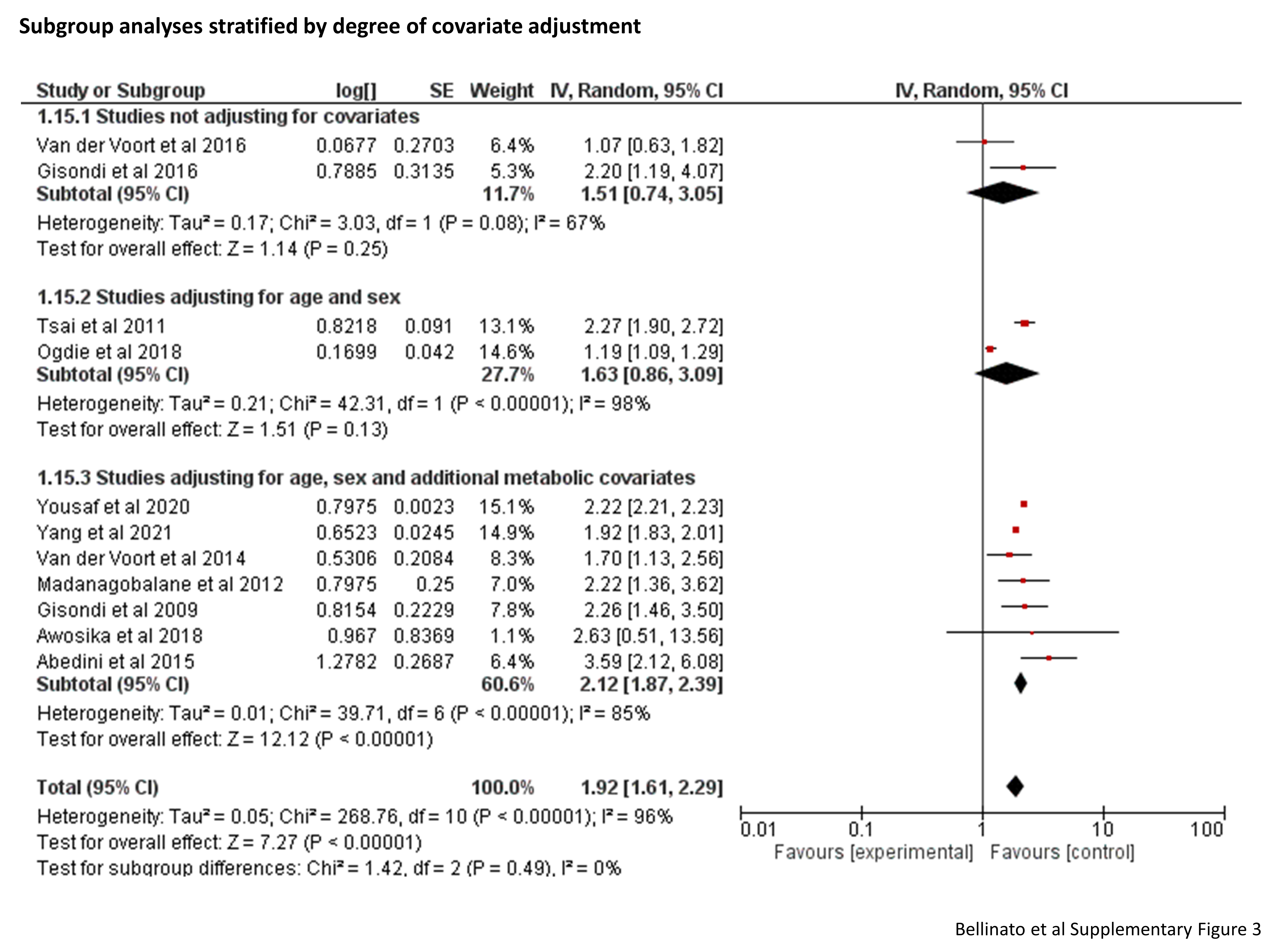

Supplement: Supplementary file 4 — Supplementary figure 3. Subgroup analysis. Forest plot and pooled estimates of the effect of psoriasis on the risk of NAFLD in 11 eligible studies, stratified by degree of covariate adjustment. (TIF 4473 kb) [file 40618_2022_1755_MOESM4_ESM.tif]

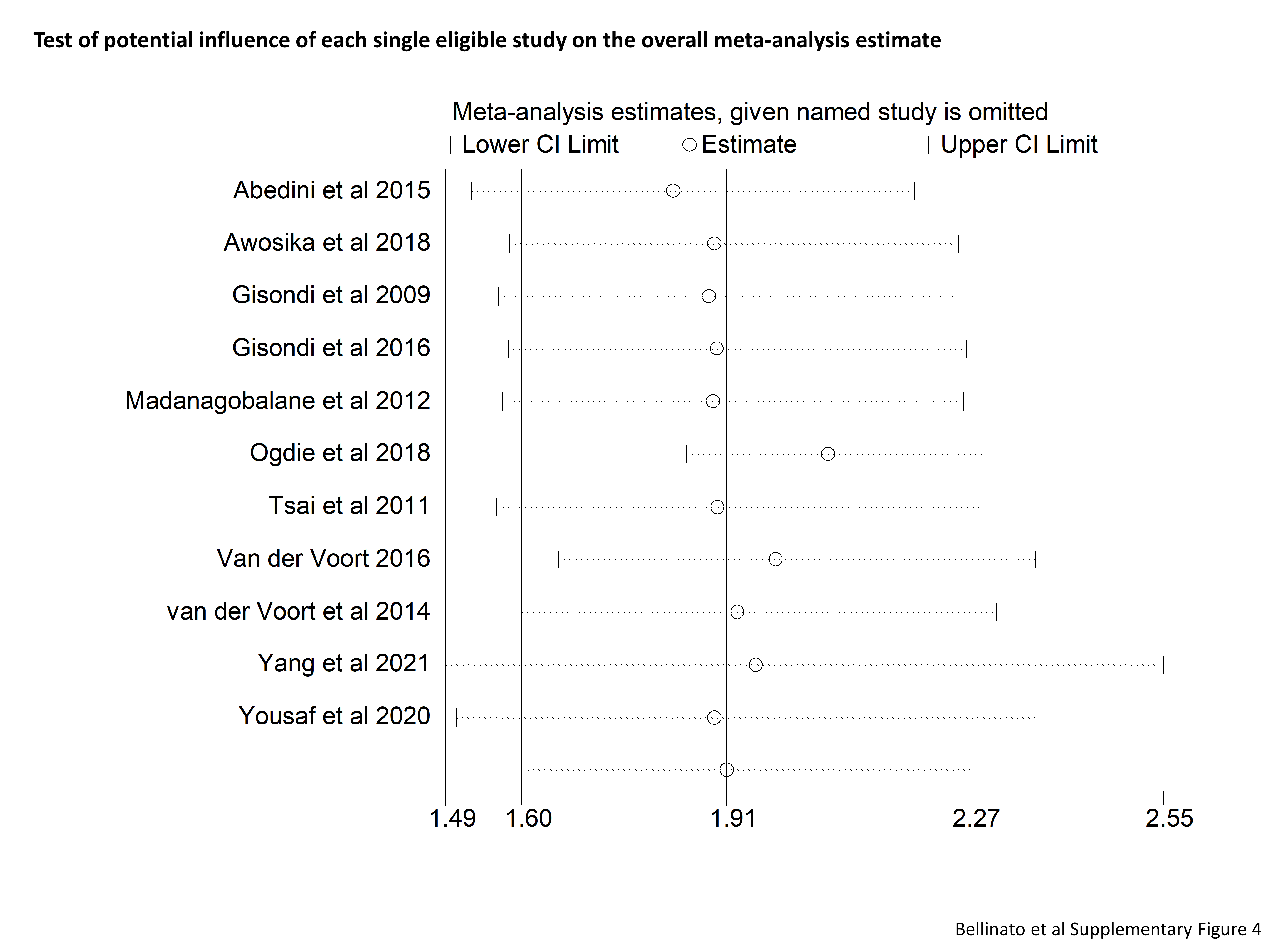

Supplement: Supplementary file 5 — Supplementary figure 4. Meta-analysis estimates, given named study is omitted (for the studies included in Figure 2). (TIF 1170 kb) [file 40618_2022_1755_MOESM5_ESM.tif]

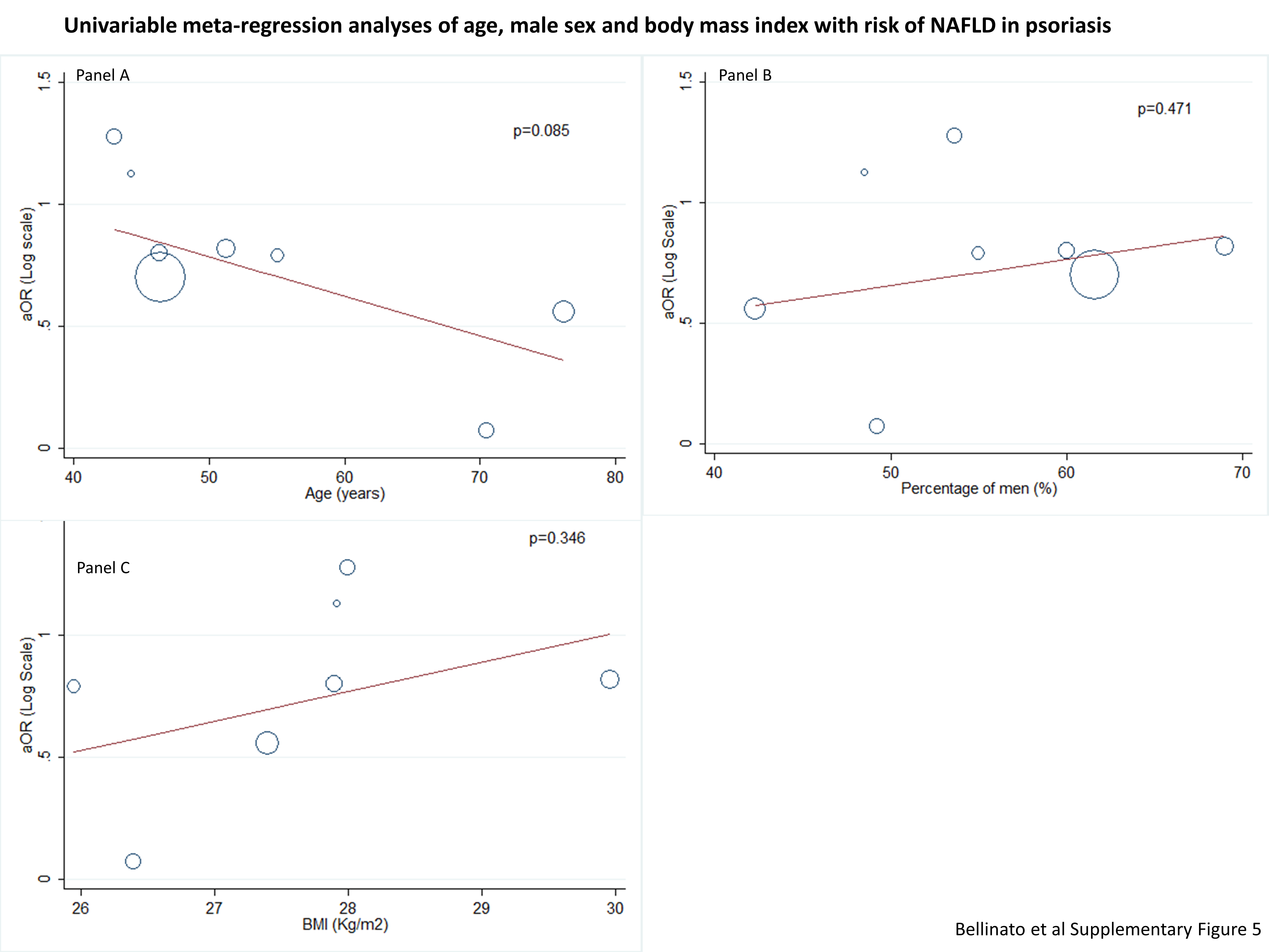

Supplement: Supplementary file 6 — Supplementary figure 5. Univariable meta-regression analyses to examine the impact of age (panel A), male sex (panel B), or body mass index (panel C) on the effect size of the risk of psoriasis-related NAFLD. (TIF 2172 kb) [file 40618_2022_1755_MOESM6_ESM.tif]

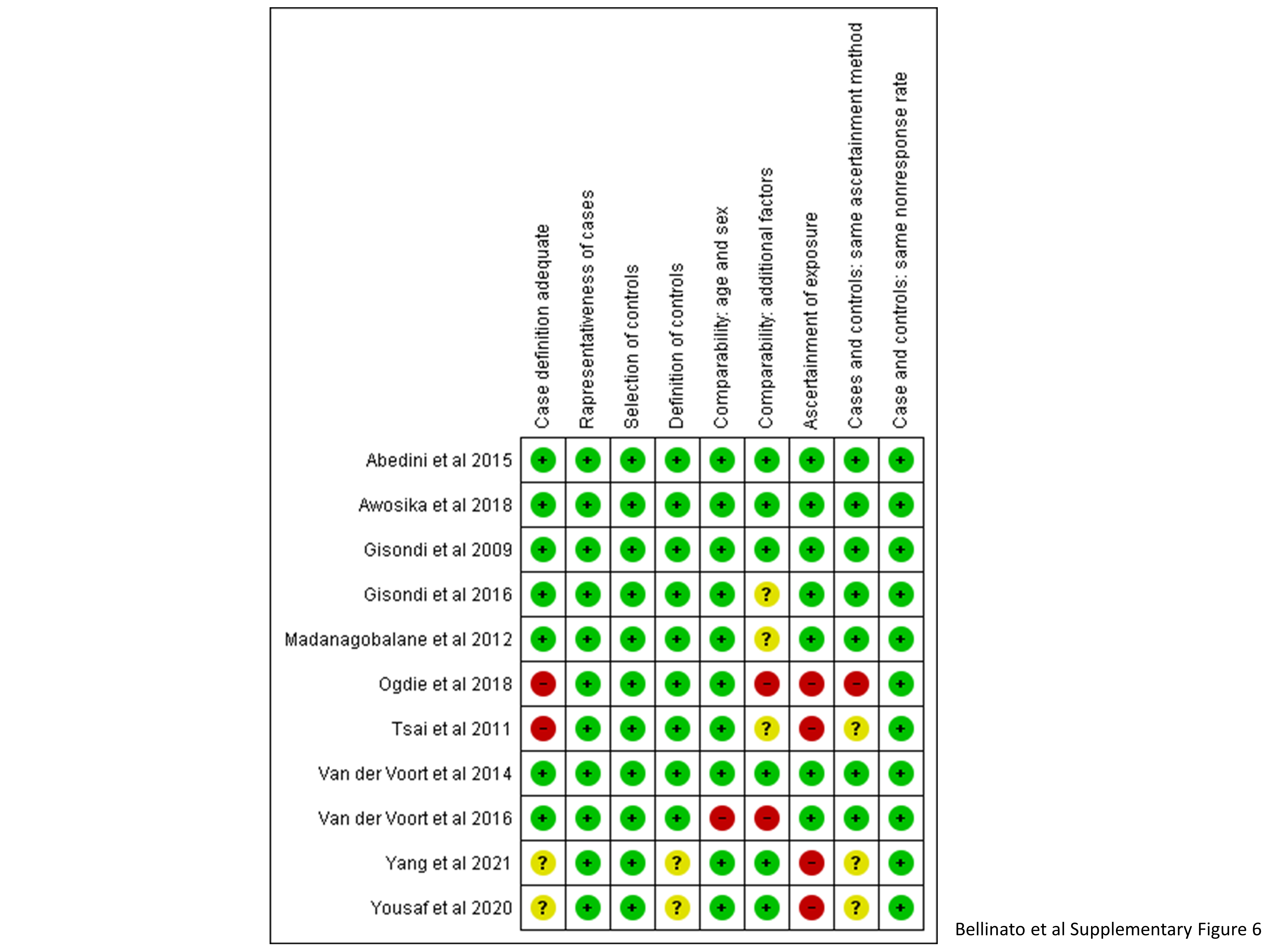

Supplement: Supplementary file 7 — Supplementary figure 6. Risk of bias summary for each eligible study assessed by the Cochrane Collaboration’s tool. (TIF 3680 kb) [file 40618_2022_1755_MOESM7_ESM.tif]

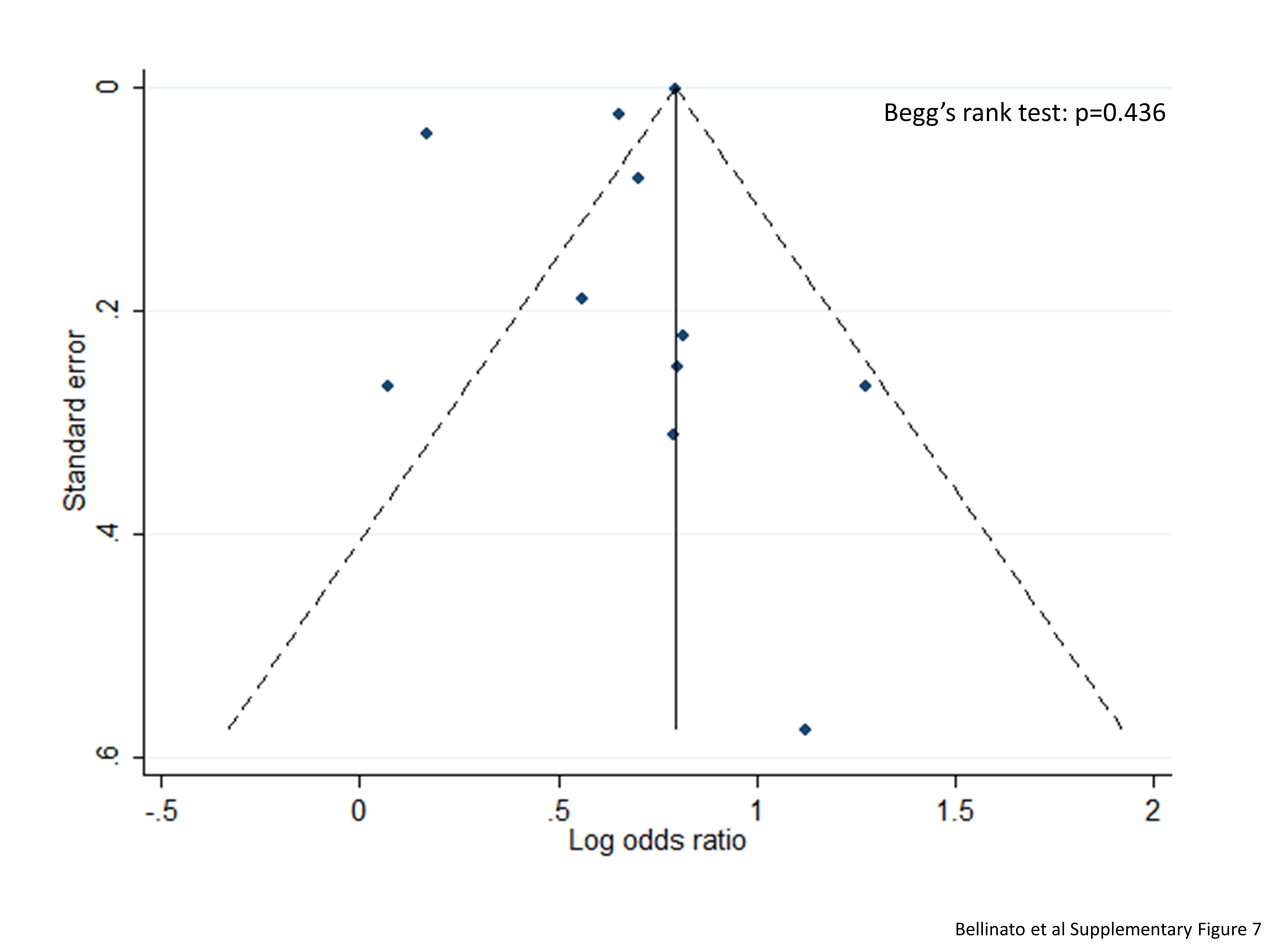

Supplement: Supplementary file 8 — Supplementary figure 7. Funnel plot of standard error by log-odds ratio for the risk of NAFLD (for the 11 eligible studies included in Figure 2). P-values by the rank correlation Begg’s test. (TIF 1848 kb) [file 40618_2022_1755_MOESM8_ESM.tif]
